# Supplementary material for: MSMCE: A novel representation module for classification of raw mass spectrometry data
Source: PLoS One. 2025 Aug 6;20(8):e0321239. doi: 10.1371/journal.pone.0321239 (PMC12327681; doi:10.1371/journal.pone.0321239)
Supplement: Attachment — Submitted filename: Response to Reviewers.docx [file pone.0321239.s004.docx]

**Response letter for “MSMCE: A Novel Representation Module for Classification of Raw Mass Spectrometry Data”**

**(Original Paper Number: PONE-D-25-08088)**

Dear Editor and Reviewers,

Thank you for your letter dated May 06, 2025, and for the opportunity to revise our manuscript titled "MSMCE: A Novel Representation Module for Classification of Raw Mass Spectrometry Data" (PONE-D-25-08088). We also extend our sincere gratitude to the reviewers for their insightful comments and constructive suggestions, which have been invaluable in improving the quality and clarity of our paper.

We have carefully considered all the points raised by the Academic Editor and the reviewers. We believe that the revisions made have substantially strengthened the manuscript. We have addressed each comment in a point-by-point manner below, and for ease of review, we have highlighted the revisions in red. The changes in the revised manuscript have been marked using the "Track Changes" feature in Microsoft Word, as requested. We have also uploaded a clean, unmarked version of the revised manuscript.

We hope that the revised manuscript is now suitable for publication in PLOS ONE.

Sincerely,

Fengyi Zhang, Boyong Gao, Yinchu Wang, Lin Guo, Wei Zhang, Xingchuang Xiong.

**CONTENTS**

[**Academic Editor**	4](#_Toc197509687)

[**<Journal Requirement #1>**	4](#_Toc197509688)

[**<Journal Requirement #2>**	4](#_Toc197509689)

[**<Journal Requirement #3>**	4](#_Toc197509690)

[**<Journal Requirement #4>**	4](#_Toc197509691)

[**Reviewer #1**	5](#_Toc197509692)

[**<Comment #1-1>**	5](#_Toc197509693)

[**<Comment #1-2>**	6](#_Toc197509694)

[**<Comment #1-3>**	6](#_Toc197509695)

[**<Comment #1-4>**	7](#_Toc197509696)

[**<Comment #1-5>**	7](#_Toc197509697)

[**<Comment #1-6>**	8](#_Toc197509698)

[**<Comment #1-7>**	8](#_Toc197509699)

[**<Comment #1-8>**	9](#_Toc197509700)

[**<Comment #1-9>**	9](#_Toc197509701)

[**<Comment #1-10>**	10](#_Toc197509702)

[**Reviewer #2**	11](#_Toc197509703)

[**<Comment #2-1>**	11](#_Toc197509704)

[**<Comment #2-2>**	11](#_Toc197509705)

[**<Comment #2-3>**	12](#_Toc197509706)

[**<Comment #2-4>**	12](#_Toc197509707)

[**<Comment #2-5>**	13](#_Toc197509708)

[**<Comment #2-6>**	14](#_Toc197509709)

[**<Comment #2-7>**	14](#_Toc197509710)

[**<Comment #2-8>**	15](#_Toc197509711)

[**<Comment #2-9>**	15](#_Toc197509712)

[**<Comment #2-10>**	16](#_Toc197509713)

[**<Comment #2-11>**	16](#_Toc197509714)

**[<Comment #2-12>](#_Toc197509715)**	[17](#_Toc197509715)

**[<Comment #2-13>](#_Toc197509716)**	[17](#_Toc197509716)

**[<Comment #2-14>](#_Toc197509717)**	[18](#_Toc197509717)

**[<Comment #2-15>](#_Toc197509718)**	[18](#_Toc197509718)

**[<Comment #2-16>](#_Toc197509719)**	[19](#_Toc197509719)

**[<Comment #2-17>](#_Toc197509720)**	[19](#_Toc197509720)

**[<Comment #2-18>](#_Toc197509721)**	[20](#_Toc197509721)

**[<Comment #2-19>](#_Toc197509722)**	[21](#_Toc197509722)

**[<Comment #2-20>](#_Toc197509723)**	[22](#_Toc197509723)

**[<Comment #2-21>](#_Toc197509724)**	[22](#_Toc197509724)

**[<Comment #2-22>](#_Toc197509725)**	[23](#_Toc197509725)

**[<Comment #2-23>](#_Toc197509726)**	[24](#_Toc197509726)

**[<Comment #2-24>](#_Toc197509727)**	[25](#_Toc197509727)

**[<Comment #2-25>](#_Toc197509728)**	[26](#_Toc197509728)

**[<Comment #2-26>](#_Toc197509729)**	[26](#_Toc197509729)

[**<Comment #2-27>**	26](#_Toc197509730)

[**<Comment #2-28>**	27](#_Toc197509731)

[**<Comment #2-29>**	27](#_Toc197509732)

[**<Comment #2-30>**	28](#_Toc197509733)

[**<Comment #2-31>**	28](#_Toc197509734)

[**Reviewer #3**	30](#_Toc197509736)

[**<Comment #3-1>**	30](#_Toc197509737)

[**<Comment #3-2>**	30](#_Toc197509738)

[**<Comment #3-3>**	31](#_Toc197509739)

[**<Comment #3-4>**	32](#_Toc197509740)

**Academic Editor**

**<Journal Requirement #1>** *Please ensure that your manuscript meets PLOS ONE's style requirements, including those for file naming.*

**<Response #1>** We have carefully reviewed PLOS ONE's style requirements and formatting templates. The manuscript has been revised to ensure full compliance with these guidelines, including file naming conventions for the submitted documents.

**<Journal Requirement #2>** *Please note that funding information should not appear in any section or other areas of your manuscript. We will only publish funding information present in the Funding Statement section of the online submission form. Please remove any funding-related text from the manuscript.*

**<Response #2>** We have ensured that all funding-related text has been removed from the manuscript body. The funding information has been solely provided in the "Funding Statement" section of the online submission form.

**<Journal Requirement #3>** *We note that the grant information you provided in the ‘Funding Information’ and ‘Financial Disclosure’ sections do not match.* *When you resubmit, please ensure that you provide the correct grant numbers for the awards you received for your study in the ‘Funding Information’ section.*

**<Response #3>** We apologize for this discrepancy. We have carefully re-checked and corrected the grant information. The 'Funding Information' section in the online submission form now accurately reflects the grant numbers for the awards received for this study, and it aligns with the 'Financial Disclosure' section.

**<Journal Requirement #4>** *Please ensure that your manuscript meets PLOS ONE's style requirements, including those for file naming.*

**<Response #4>** Thank you for the reminder. We have made our best effort to revise the manuscript in accordance with PLOS ONE's style requirements, including those for file naming. If there are any further specific style or formatting modifications needed, please do let us know.

**Reviewer #1**

**<Comment #1-1>** *1. While performance metrics (accuracy, precision, recall, F1) are thoroughly reported, the manuscript lacks statistical testing to validate the significance of the observed improvements. Repeated runs with different random seeds and the use of standard tests (e.g., Wilcoxon signed-rank test, bootstrapping confidence intervals) are recommended. This is particularly important for small-sample settings (e.g., Canine Sarcoma dataset).*

**<Response #1-1>** Thank you for this important suggestion regarding statistical validation of our results. We have addressed this by incorporating more rigorous statistical analysis in our revised manuscript.

Specifically:

1. Our experimental design, as detailed in Section 'Experimental Setup and Training Strategy', already employs a k-fold stratified cross-validation (with k=6]) where each model is trained k times on different folds of the training data and evaluated on a common hold-out test set. This provides multiple performance measurements for each model on the test set.
2. Bootstrap Confidence Intervals: We now report the 95% bootstrap confidence intervals for the mean performance metrics (Accuracy, Precision, Recall, F1-score) obtained from these k-fold evaluations on the hold-out test set. These confidence intervals provide a measure of the uncertainty around our reported mean values and are summarized in the table provided in the Supporting Information (Supporting File S3).
3. Wilcoxon Signed-Rank Test for Paired Comparisons: To assess the statistical significance of the improvements observed when using our MSMCE module compared to baseline models (i.e., models without MSMCE), we have performed paired Wilcoxon signed-rank tests. These tests compare the performance metrics (e.g., accuracy) of the MSMCE-enhanced model versus its corresponding baseline model across the k test set evaluations from the k-fold cross-validation. The resulting p-values are now reported in the text when discussing these comparisons in the **'Experiment and Results' section** (e.g., "MSMCE-ResNet50 showed a statistically significant improvement in accuracy over the baseline ResNet-50 (p = 0.03125)").

While we used a fixed global random seed for reproducibility in the current k-fold cross-validation framework, the use of k-fold itself provides multiple evaluation points. We believe these additions, particularly the inclusion of bootstrap confidence intervals and Wilcoxon signed-rank test results, now provide the necessary statistical validation for the observed performance improvements, especially for datasets like Canine Sarcoma.

**<Comment #1-2>** *2. The MSMCE module's effectiveness is well demonstrated quantitatively, but the biological meaning of the learned features is not explored. Given the biomedical context of mass spectrometry data, the manuscript would benefit from a discussion (or illustrative example) of how MSMCE embeddings relate to biologically meaningful features or clinically relevant patterns. This is important for building trust and enabling adoption in translational research.*

**<Response #1-2>** We sincerely thank the reviewer for this insightful comment and for highlighting the importance of exploring the biological meaning of the learned features, especially given the biomedical context of mass spectrometry data. We agree that understanding how MSMCE embeddings might relate to biologically significant features or clinically relevant patterns is crucial for building trust and facilitating translational research.

In the current study, our primary objective was to investigate, from a computational and data science perspective, a novel feature representation methodology for raw mass spectrometry data. Specifically, we focused on the development and empirical validation of the MSMCE and its impact on the classification performance and training efficiency of deep learning models. Our research aimed to determine whether transforming single-channel MS data into a multi-channel representation could provide tangible benefits for model training and predictive accuracy, as demonstrated by our quantitative results across four public datasets.

Therefore, while our current work establishes the computational advantages of the MSMCE module, we consider the interpretation of its learned features in a biological or clinical context as an important avenue for future research. We have now explicitly addressed this point in the revised manuscript by expanding the **'Limitations and future directions' subsection within the 'Discussion' section**. In this revised section, we clearly state that uncovering the biological significance of the MSMCE embeddings is a key area for subsequent investigation.

We appreciate the reviewer's guidance in emphasizing this aspect, as it will undoubtedly shape our future work in this domain.

**<Comment #1-3>** *3. The manuscript refers to a “residual connection” via channel concatenation, which is misleading, as no additive operation is applied. Please revise the terminology to avoid confusion with standard residual connections.*

**<Response #1-3>** We sincerely thank the reviewer for this precise and important observation. We acknowledge that our use of the term 'residual connection' in the context of channel concatenation could be misleading, as it does not involve the additive operation characteristic of standard residual connections found in architectures like ResNet. Our intention was to describe an operation that similarly aims to preserve and pass through earlier-stage features (the globally encoded feature $E^{'}$) alongside newly transformed features (the multi-channel convolutional embeddings $C_{2}$).

To address this and ensure clarity, we have revised the manuscript to more accurately describe this operation. Specifically:

1. We have removed the term 'residual connection' where it might cause confusion with additive skip connections.
2. We now describe the operation explicitly as 'feature concatenation along the channel dimension to integrate global and local embeddings.'
3. The purpose of this concatenation—to combine the initial globally encoded representation $E^{'}$ (with shape $B\times1\times d$) with the locally refined multi-channel convolutional features $C_{2}$ (with shape $B\times C\times D$) to form a richer, combined representation $O$ (with shape $B\times\left( 1+C \right)\times d$)—is now stated more directly without relying on the 'residual' analogy.

These changes have been implemented in the **Abstract, Introduction, the 'Materials and Methods' section (specifically the subsection previously titled 'Channel “Residual” Connection', which has been renamed, and in its description)**, and the Discussion. We believe these revisions accurately reflect our methodology and prevent any potential misunderstanding with standard additive residual connections.

**<Comment #1-4>** *4. Details about hyperparameter selection, learning rate tuning, and batch sizes should be more explicitly stated or summarized in a table.*

**<Response #1-4>** Thank you for this comment. We have ensured that details regarding hyperparameter selection and learning rate tuning are explicitly stated within the revised **'Experimental Setup and Training Strategy' section** of the revised manuscript. Furthermore, the batch sizes used for the experiments are introduced in the preliminary description of the **'Experiment and Results' section** (prior to detailing the dataset-specific results, of the revised manuscript). We believe these sections now provide the necessary clarity on these parameters.

**<Comment #1-5>** *5. The methods for computing FLOPs and model size should be clearly described.*

**<Response #1-5>** Thank you for requesting clarification on the methods used to compute FLOPs and model size. We have added these details to the **'Computational Efficiency Analysis' subsection within the 'Experiment and Results' section** of our revised manuscript.

Specifically:

1. FLOPs (Floating Point Operations): FLOPs were calculated using the thop library. This calculation was performed by providing a single sample tensor with dimensions (1, spectrum_dim) (where spectrum_dim is the length of the binned mass spectrum before MSMCE processing) as input to the profile function. FLOPs are reported in GigaFLOPs (GFLOPs).
2. Model Size (Number of Parameters): The 'model size' reported in our study refers to the Estimated Total Size (MB) of GPU memory occupied by the model during one forward and one backward pass. This was obtained using the torchinfo.summary function, with an input size corresponding to the batch size used during training (i.e., (batch_size, spectrum_dim)). This metric provides an estimate of the peak memory footprint during training.

**<Comment #1-6>** *6. The t-SNE plots and radar charts in the supplementary material are helpful but not discussed in the main text. Please integrate interpretations of these visualizations into the Results or Discussion to show how MSMCE improves feature separability and efficiency.*

**<Response #1-6>** Thank you for this valuable suggestion. We agree that discussing these visualizations in the main text will enhance the manuscript.

1. t-SNE Visualizations: We have now incorporated a discussion of the t-SNE visualizations (provided in the supplementary material, illustrating the data distributions for each dataset) into the **preliminary description of the 'Experiment and Results' section**. This addition aims to provide an initial qualitative insight into the class separability of the datasets before the application of our models. While these t-SNE plots are based on the original processed data features (prior to MSMCE embeddings) and thus do not directly show how MSMCE improves feature separability, they offer a baseline understanding of the dataset’s characteristics.
2. Radar Charts: We would like to clarify that the radar charts, which illustrate model efficiency (FLOPs, model size) in relation to accuracy, are already discussed in detail within the **'Computational Efficiency Analysis' subsection of the 'Experiment and Results' section**. This discussion explicitly highlights how MSMCE improves computational efficiency.

**<Comment #1-7>** *7. The manuscript is well-written in general. Minor edits are suggested for typographical consistency (e.g., consistent table formatting, figure references). Also, be precise with terms such as "residual connection".*

**<Response #1-7>** We appreciate the reviewer's positive feedback on the overall writing. We have carefully reviewed the entire manuscript and made edits to ensure typographical consistency, including table formatting and figure references. Furthermore, we have taken care to use terminology precisely. For instance, as per earlier feedback and the reviewer's note, the term analogous to 'residual connection' which is achieved via channel concatenation has been revised to more accurately describe the operation as 'channel concatenation', avoiding any misleading association with standard additive residual connections.

**<Comment #1-8>** *8. The references are not cited in sequential order (e.g., the citation sequence jumps from [3–8] to [10], or from [24–26] to [22,32,39]), which may confuse readers and should be corrected for consistency.*

**<Response #1-8>** Thank you for pointing out the inconsistencies in our citation order. We have thoroughly reviewed and revised the manuscript to ensure that all references are now cited in strict numerical sequence according to their first appearance in the text, and that the reference list is ordered accordingly. This has been corrected throughout the manuscript for consistency.

**<Comment #1-9>** *9. Although ReLU and Dropout are standard components in deep learning, they should be briefly explained for clarity, given that PLOS ONE targets a multidisciplinary audience that may not be familiar with these concepts.*

**<Response #1-9>** We thank the reviewer for this thoughtful suggestion. We agree that providing brief explanations for standard deep learning components like ReLU and Dropout is beneficial for enhancing the clarity and accessibility of our manuscript to a multidisciplinary readership, which PLOS ONE serves.

In the revised manuscript, we have incorporated concise definitions and the purposes of both ReLU and Dropout. These explanations have been added to the **'Materials and Methods' section, within the subsection 'Fully Connected Encoder'**, where these components are first introduced as part of our MSMCE module's architecture. We believe this will help readers who may not be deeply familiar with these specific deep learning techniques to better understand their roles within our proposed model. Specifically, we have clarified that:

1. ReLU (Rectified Linear Unit) is an activation function that introduces non-linearity into the model, outputting the input directly if it is positive, and zero otherwise, which helps with issues like vanishing gradients and computational efficiency.
2. Dropout is a regularization technique used during training where a proportion of neuron outputs in a layer are randomly ignored, which helps prevent overfitting and improves the model's generalization to unseen data.

**<Comment #1-10>** *10. Figures 1 to 3 are of very poor quality, with unreadable text. They should be replaced with higher-resolution versions to ensure legibility and clarity.*

**<Response #1-10>** We apologize for the issues with the figure quality experienced by the reviewer. We believe this may be due to the compression of images when the journal generates the review manuscript. We had initially submitted high-resolution figures. For this resubmission, we have re-checked and ensured that Fig 1 to 3 (and all other figures) are provided in high resolution, meeting PLOS ONE’s image guidelines, to ensure their legibility and clarity. We have also utilized the PACE tool as recommended to verify figure compliance.

**Reviewer #2**

**<Comment #2-1>** *1. The last sentence of the abstract: “Experimental results ...MS data classification.” The link between reduced computational resource and generalizability of the model is not given and trivial.*

**<Response #2-1>** We thank the reviewer for pointing out the need for greater clarity regarding the link between reduced computational resources/enhanced training efficiency and model generalizability in the abstract. We agree that this connection is not direct or self-evident and that our original phrasing might have inadvertently implied a direct causal relationship.

Our intention was to highlight that while the primary contributions of MSMCE are significant improvements in classification performance and computational efficiency, the latter (enhanced efficiency) can indirectly facilitate the development of more generalizable models. For instance, reduced training time and lower resource consumption allow for more extensive hyperparameter tuning, cross-validation, and experimentation with different model architectures or larger datasets, all of which are crucial for building models that generalize better to unseen data.

However, to avoid any misinterpretation and to maintain a precise focus on the direct, demonstrated benefits, we have revised the concluding part of the abstract. The revised sentence now emphasizes the demonstrated effectiveness in classification and efficiency, and more cautiously suggests its potential for broader applicability, without making a strong, unproven claim about directly enhancing generalizability due to resource reduction.

The revised sentence in the abstract now reads: ' Experimental results on four public datasets demonstrate that the proposed MSMCE module not only achieves substantial improvements in classification performance but also enhances computational efficiency and training stability, highlighting its effectiveness in raw MS data classification and its potential for robust application across diverse datasets.'

**<Location #2-1>** Abstract section, final sentence.

**<Comment #2-2>** *2. This sentence in the introduction: “The large ... cancer detection”. The challenges need to be explicitly included. Explicitly including the challenges help to develop motivation of the paper.*

**<Response #2-2>** We thank the reviewer for this valuable suggestion. We agree that explicitly listing the challenges associated with MS data analysis in the introduction will better establish motivation for our work.

In the revised manuscript, we have expanded the sentence referenced by the reviewer to more clearly enumerate these challenges. The revised sentence now highlights not only the large volume and high dimensionality but also other critical issues such as the inherent noise, signal variability (e.g., peak shifts and intensity fluctuations).

**<Location #2-2>** Introduction section, first paragraph.

**<Comment #2-3>** *3. This sentence: “These operations ... MS signal acquisition,” the issues need to be explicitly listed: such as peak shift, etc.*

**<Response #2-3>** Thank you for this suggestion. We agree that explicitly listing common issues encountered during MS signal acquisition will improve clarity. In the revised paragraph within the Introduction section (now the second paragraph), we have expanded on the 'issues arising during MS signal acquisition' to include specific examples such as 'peak shifts across spectra, baseline drift, noise interference, and variations in signal intensity,' which these preprocessing operations aim to correct.

**<Location #2-3>** Introduction section, second paragraph.

**<Comment #2-4>** *4. This sentence: “Nevertheless, the high dimensionality of MS data combined with limited sample sizes makes it challenging for traditional methods to effectively meet the demands of data analysis [11].”, It is not the high dimensionality of data or small sample size that limits the performance of the traditional methods, but the mismatches between bathes and the need for preprocessing of data so the methods work. Actually, deep learning models need more data to be trained on compared to conventional methods especially if the data has higher complexity and dimensions. Reference 11 is not correctly referenced as it talks about using matrix factorization and using Bayesian framework to overcome such problems and does not explicitly relay the logic used in this sentence. Regardless, the sentence is not following logic and message of this paragraph.*

**<Response #2-4>** We appreciate the reviewer's detailed feedback on this sentence and the use of Reference [11]. We acknowledge the reviewer's point that factors like batch effects and the necessity of extensive preprocessing also significantly challenge traditional methods, and that deep learning models often require substantial data. Our intention was not to overlook these aspects but to highlight a widely recognized challenge in omics data analysis, which is also pertinent to traditional machine learning approaches when applied to MS data.

Regarding Reference [11] (Niu et al., "Deep learning framework for integrating multibatch calibration, classification, and pathway activities"), while the main focus of that paper is indeed on a deep learning framework for batch effect correction and other tasks, the introduction of their paper (specifically the third paragraph, as we re-checked) does state: 'It is still a challenge for machine learning... to perform computational analysis, considering many measurements (typically referring to the feature dimensionality) and usually a limited number of samples in high-throughput omics data.' This statement from Reference [11] supports our assertion that the combination of high dimensionality and limited samples poses a challenge for machine learning in general, which includes traditional methods when applied to complex omics data like mass spectrometry. Our sentence aimed to reflect this general challenge which sets a context for why advanced methods (including, eventually, deep learning approaches like ours) are explored.

However, we understand the reviewer's concern that our original sentence might have oversimplified the limitations of traditional methods. We also agree that the overall flow of the paragraph could be improved. To address these points, we have revised the paragraph in the Introduction section (currently the second paragraph of the Introduction). The revised text now aims to:

1. Acknowledge more explicitly that traditional methods heavily rely on preprocessing to handle issues like batch effects and noise.
2. Rephrase the statement about high dimensionality and limited samples to be more nuanced, framing it as one of several factors that can make it difficult for traditional methods to fully exploit the rich information in MS data without meticulous feature engineering and optimization, especially when compared to the potential of end-to-end learning approaches.
3. Ensure that the reference to [11] is appropriately contextualized, perhaps by focusing the sentence more broadly on the general challenges of omics data that traditional machine learning faces, which is supported by the introduction of Niu et al.
4. Smooth the transition to the subsequent discussion on deep learning.

We believe these changes, along with a careful re-reading of the entire paragraph for logical flow, address the reviewer’s concerns more comprehensively. We also re-verified other citations in this paragraph to ensure their relevance.

**<Location #2-4>** Introduction section, second paragraph.

**<Comment #2-5>** *5. This sentence: “These methods often fail to fully exploit the latent information within the data, thereby limiting their overall performance.”. No reference. This claim is not accurate and somewhat controversial. The concern is: these methods (if used correctly) are robust as cited in the same paragraph, but preprocessing data to feed to these models require careful fine tunings. Needs to be rewritten, maybe restructuring like: Achieving optimal performance with these methods typically involves multiple stages of preprocessing and fine-tuning of model parameters to fully leverage the rich, latent information embedded in complex MS data.*

**<Response #2-5>** Thank you for this constructive criticism and the excellent suggestion for rephrasing. We agree that the original statement was too strong and could be seen as contradictory. We have adopted the reviewer's recommended direction. The revised paragraph in the Introduction section now reflects the idea that achieving optimal performance with traditional methods indeed requires careful and often complex preprocessing and parameter tuning to effectively harness the latent information in MS data. We have removed the contested phrase and integrated a more nuanced perspective that acknowledges the potential robustness of these methods when meticulously applied, while still highlighting the intensive effort this can entail, which motivates the exploration of methods with more automated feature learning capabilities.

**<Location #2-5>** Introduction section, second paragraph.

**<Comment #2-6>** *6. This sentence:” In recent years, deep learning has emerged as a dominant technology”. Deep learning is not a technology, rather an approach or methodology.*

**<Response #2-6>** We thank the reviewer for this precise correction regarding the terminology used for deep learning. We have revised the manuscript accordingly. The term 'technology' has been replaced with 'approach' in the **Abstract and in the Introduction section** of the revised manuscript.

**<Comment #2-7>** *7. The term channel in this article has been used in title and as a cornerstone of the article. For the first-time reader, the term is confusing, because, in the context of MS data, channel does not have a single universal definition. The authors are using channel as a concept of feature representations from CNN layers. This becomes clear later in the methods section. The term channel needs to be clarified and defined explicitly in the introduction. There is also some mix-up of term channel and concept of dimension. For example, in this sentence: “In the field of image classification, multi-channel images contain complementary information across different channels, enabling a more comprehensive description of the target compared to single-channel representations”. Consider confusion in this sentence: “These multi-channel features not only enrich feature representations but also enhance the model’s adaptability to high-dimensional data.” I suggest definition of term “channel” in the introduction, and then being careful not to use it in interchange with dimension- representation- feature-etc.*

**<Response #2-7>** Thank you for this very helpful feedback on the clarity and use of the term 'channel.' We agree that an early and explicit definition is crucial.

In the revised manuscript, we have addressed this by:

1. Introducing a channel description after the sentence: “In the field of image classification…” that 'channels' typically refer to color channels.
2. Immediately following this analogy, we explicitly define 'channel' as used in our work: 'In our work, a 'channel' specifically refers to a distinct feature map produced by a convolutional layer. Each such channel embodies a learned embedding representation or a filtered perspective of the input data.'
3. We have also carefully revised the sentence previously highlighted by the reviewer, "These multi-channel features not only enrich feature representations but also enhance the model’s adaptability to high-dimensional data”, to more precisely articulate how the structured multi-channel output aids in processing the original high-dimensional MS input, thereby avoiding confusion between 'channels' and the 'dimensionality' of the raw data.
4. Furthermore, we have reviewed the entire manuscript to ensure consistent use of 'channel' as defined and to distinguish it clearly from 'dimension' and other related terms.

**<Comment #2-8>** *8. This sentence does not read well: MS2DeepScore [25] employs a Siamese neural network to learn low-dimensional embeddings of MS vectors for predicting the structural similarity between chemical compounds, which is also applicable to MS Clustering. Maybe a comma is missing after vectors?*

**<Response #2-8>** Thank you for pointing out the lack of clarity in this sentence. We have restructured the latter part of the sentence slightly to improve its flow and clarity.

**<Comment #2-9>** *9. In this sentence: Studies have shown that deep learning can directly capture complex patterns”, what is the implication of the word “directly”? What would the indirect way be? I suggest removing the word “directly”.*

**<Response #2-9>** Thank you for this suggestion. We used the word 'directly' to emphasize that deep learning models can learn features from raw or minimally processed data without extensive manual feature engineering, which is often required by more traditional machine learning methods (the 'indirect' way). However, we agree with the reviewer that the term might be ambiguous or unnecessary here. We have removed the word 'directly' from this sentence in the Introduction section of the revised manuscript with track changes.

**<Comment #2-10>** *10. deep classification model” is not a valid term.*

**<Response #2-10>** Thank you for pointing this out. We have revised the manuscript to use more standard and precise terminology. Instances of 'deep classification model' have been replaced with 'deep learning-based classification model,' 'deep learning classifier,' or by referring to the specific model architectures used (e.g., CNN, LSTM, Transformer) throughout the text.

**<Comment #2-11>** *11. In the section where authors outline the contribution of this study:“End-to-End Training Framework”, training the DL+classifier model in an end-to-end manner is not innovative and is widely used. See: Seddiki, Khawla, et al. "Early diagnosis: End-to-end CNN–LSTM models for mass spectrometry data classification." Analytical Chemistry 95.36 (2023): 13431-13437.*

**<Response #2-11>** We thank the reviewer for this comment and for providing the relevant reference. Our work is also inspired by this work.

Our intention in listing "End-to-End Training Framework" as a contribution was not to claim novelty for the end-to-end training methodology itself, but rather to highlight a key characteristic and strength of our proposed MSMCE module: its design as a learnable representation module that can be seamlessly integrated as a 'front-end' to various existing deep learning classifiers to form a unified, end-to-end trainable system. The contribution lies in MSMCE’s ability to transform raw MS data into an enhanced multi-channel representation that is directly fed into these classifiers, allowing for joint optimization of both the representation learning and the classification task. This facilitates the automatic learning of optimal features tailored to the specific downstream classifier and dataset.

To clarify this, we have revised the description of this contribution point. We have rephrased it to emphasize the integrability of the MSMCE module within an end-to-end learning context, rather than the end-to-end framework itself as the novelty. The revised phrasing focuses on how MSMCE enables and enhances end-to-end learning for MS data classification by providing a learnable, optimized input representation for diverse classifiers. We believe this better reflects the specific contribution of MSMCE in this regard.

**<Comment #2-12>** *12. “Dimensional Adaptation of Multi-Channel Embedding Representations:” This contribution is not clear. It is not clear what authors mean by “adaptability”. “Compared to the original single-channel MS vectors, the embedded multi-channel vectors exhibit better adaptability within CNN architectures.”, how has the adaptability been quantified? What are the statistics of the enhanced adaptability, where is the pvalue?*

**<Response #2-12>** Thank you for your feedback regarding the clarity of the previously stated contribution on 'Dimensional Adaptation.' We acknowledge that the term 'adaptability' was not clearly defined or quantitatively substantiated. Our intention was to convey that the structured multi-channel output of MSMCE (with a shape like $B\times\left( 1+C \right)\times d$) is dimensionally more aligned with the typical input expectations of convolutional and sequence models compared to the original, very high-dimensional single-channel MS vectors.

To address this, we have removed that specific contribution point and replaced it with a more concrete and empirically supported contribution: 'Enhanced Computational Efficiency and Training Stability.' This revised contribution, detailed in the 'Contributions' subsection of the Introduction, is directly supported by quantifiable metrics such as reduced FLOPs and memory footprint for CNNs, and improved training stability for sequence models, as presented in our 'Computational Efficiency Analysis' section (Figure 5) and throughout the results. We believe this change makes our contributions clearer and more robustly demonstrated.

**<Comment #2-13>** 1*3. A general feedback to the authors. Authors have used a fully connected layer as the first layer. It is not wrong and authors’ choice to do so, but in my opinion, it is counter intuitive to use a flat layer to reduce dimensionality. The local MS information is lost this way. When transforming using WX+b, all local dependence is lost (for example peak shape, etc). This is counter-intuitive with using the CNN in the rest of the structure of the model. In most of the cited papers, usually a CNN layers comes first ensuring capturing the local dependencies of peaks, etc. But again, the proposed model by the authors is valid.*

**<Response #2-13>** We appreciate the reviewer's insightful feedback regarding our choice of a fully connected layer as the initial layer for dimensionality reduction. We understand the perspective that this might seem counter-intuitive compared to starting with convolutional layers for capturing local dependencies, and we acknowledge the potential for loss of some local MS information with an initial flattening transformation.

Our design choice for the MSMCE module's encoder was indeed based on a combination of empirical exploration and the aim to create a compact global representation of the high-dimensional MS vector before proceeding to the channel embedding stage with 1D convolutions. While alternative initial layers like 1D CNNs could also be considered, our experiments across multiple datasets and downstream classifiers (as shown in Tables 2-7 and Figure 5) consistently demonstrated that the proposed architecture, beginning with this fully connected encoder, yields significant improvements in classification performance and computational efficiency.

We value the reviewer's observation, and it highlights an interesting area for potential future comparative studies on different initial embedding strategies. For the current work, the empirical effectiveness of the presented design was the primary driver. We thank the reviewer for acknowledging the validity of our proposed model.

**<Comment #2-14>** 14*. E is not a “feature matrix” in the classic sense (i.e., not raw features you designed or extracted). It is more accurate to call it: A latent embedding, or A learned representation.*

**<Response #2-14>** We thank the reviewer for this precise and helpful terminological correction. We agree that 'feature matrix' is not the most accurate term for $E$in this context, as it represents a learned, dense representation rather than handcrafted features.

In the revised manuscript, we have replaced 'feature matrix' with more appropriate terms such as 'embedding representation,' or 'latent embedding representation' when referring to $E$. For instance, the sentence describing $E$in the **'Materials and Methods' section, under the subsection 'Fully Connected Encoder'**, has been modified to reflect this. We have also reviewed other instances where $E$ or similar learned outputs are described to ensure consistent and accurate terminology.

**<Comment #2-15>** *15. Figure 1: input is BxD but in the figure it is illustrated as 1-D.*

**<Response #2-15>** We thank the reviewer for this observation regarding the illustration of the input in Figure 1.

Figure 1 was intentionally designed to illustrate the transformation process for a single MS vector (i.e., one sample with dimension D) through the MSMCE module. The rationale for this simplified 1-D representation of the input in the figure was to enhance clarity and focus on the core architectural transformations—from a single high-dimensional vector through the encoder, into the channel embedding stage, and finally to the multi-channel output. Illustrating the full $B\times D$ batch input might have cluttered the diagram and obscured the step-by-step processing of an individual data instance.

However, we understand that this can be misleading if not explicitly stated. To address this, we have added a clarification in the caption of Figure 1 to explicitly state that the diagram illustrates the processing pathway for a single input MS vector for conceptual clarity, while the model actually processes data in batches.

**<Comment #2-16>** 1*6. Addition of channel dimension is poorly shown in the figure 1 making it harder to understand. The green input vector (which says embeding_dim) directly goes into 1D convolution skipping adding a dimension to.*

**<Response #2-16>** Thank you for pointing this out. To clarify the addition of the channel dimension in Figure 1, we have made the following revisions:

1. The output of the Encoder module (the global embedding) is labeled with its dimension $(embedding\_dim)$.
2. We have now explicitly indicated a 'Reshape' operation (via a label on the connecting arrow) before this global embedding enters the first 1D convolutional layer.
3. The reshaped representation, which serves as the input to the 1D convolution, is now clearly labeled with dimensions that include the added channel $(1, embedding\_dim)$.

**<Comment #2-17>** *17. The convolutions are 1D, and the Eprime is Bx1xd, what is “3” in dimension of K1 (1xC/2x3), which dimension the convolution is happening? The same question for K2. It seems the notion and the mapping between the size of K1 and K2 and the output is inconsistent.*

**<Response #2-17>** We sincerely thank the reviewer for meticulously pointing out this critical inconsistency in our description of the 1D convolution kernel dimensions and their relationship with the input/output tensors. We apologize for this error and the confusion it caused. The reviewer is correct that our previous notation for $K_{1}$ and $K_{2}$ was inconsistent with standard Conv1D kernel definitions (e.g., in PyTorch: out_channels, in_channels, kernel_size) and the stated input/output dimensions.

We have now corrected the description of the kernel dimensions and clarified the convolution process in the **'Materials and Methods' section, under the 'Channel Embedding Module' subsection**. The corrected understanding and description are as follows:

1. The input to the first Conv1D layer is $E'$ (referring to the reshaped global embedding) with dimensions $(in\_channels, embedding\_dim)$, where $in\_channels=1$ (the single channel from reshaping) and $embedding\_dim=d$.
2. The first convolution kernel $K_{1}$, has dimensions $(out\_channels, in\_channels, kernel\_size)$.
   a) $out\_channels=\frac{C}{2}$ (producing $\frac{C}{2}$ output channels for $C_{1}$).
   b) $in\_channels=1$ (matching the input $E'$).
   c) $kernel\_size=3$ (this is the "3" the reviewer asked about; it is the length of the 1D filter).
3. The output $C_{1}$ thus has dimensions $(\frac{C}{2}, d')$, where $d'$ would be $d$ if padding is 'same', or slightly less if no padding. (Our text stated $C_{1}\in R^{B\times\frac{C}{2}\times d}$, implying same padding or careful handling to maintain length $d$).
4. The second convolution kernel $K_{2}$, dimensions $(out\_channels, in\_channels, kernel\_size)$.
   a) $out\_channels=C$ (producing C output channels for $C_{2}$).
   b) $in\_channels=\frac{C}{2}$ (matching the input $C_{1}$).
   c) $kernel\_size=3$ (again, the length of the 1D filter).
5. The output $C_{2}$ thus has dimensions $(C, d'')$. (Our text stated $C_{2}\in R^{B\times C\times d}$, implying same padding or handling to maintain length $d$).

We believe these corrections now accurately and consistently describe the 1D convolutional operations and kernel dimensions. We apologize again for the oversight.

**<Comment #2-18>** *18. “By transforming feature vectors into multi-channel representations, this module enhances adaptability for downstream classification tasks.” this claim needs to be proven in a quantifiable way. It is not clear what does adaptability mean.*

**<Response #2-18>** Thank you for highlighting this sentence and requesting further clarification on what we mean by 'adaptability' and how it is supported.

Our intention with this statement was to convey that the structural transformation performed by the Channel Embedding module—converting a global embedding into a multi-channel representation (e.g., with shape $B\times C\times d$)—results in an output format that is more dimensionally aligned with the typical input expectations of various downstream deep learning classifiers, such as CNNs (which expect channelized input for their convolutional filters) and sequence models like LSTMs or Transformers (which can process the channels as sequences). This dimensional alignment can contribute to more effective processing by these models.

While 'suitability' in this specific context is not a directly quantifiable metric with a p-value, its benefits are indirectly evidenced by:

1. Improved Performance: The consistent and significant improvements in classification accuracy and F1-scores across diverse datasets and multiple downstream model architectures when using MSMCE suggest that the representations generated by MSMCE are indeed more effectively utilized by these classifiers.
2. Enhanced Training Stability and Efficiency: As discussed in our 'Computational Efficiency Analysis' and 'Training Process' sections, for certain models like LSTMs and Transformers, the MSMCE module not only improved performance but also enhanced training stability (preventing crashes observed with direct high-dimensional input). Furthermore, for CNNs, it led to significant reductions in FLOPs and model size. These improvements suggest that the transformed representation is better suited to the operational characteristics of these models.

We have revised the sentence in the **'Materials and Methods' section, at the end of the 'Channel Embedding Module' subsection**, it now reads: ' It transforms global embedding representation into multi-channel embedding representation, thereby providing a structured format that is dimensionally more aligned with downstream classification tasks.'

We believe this rephrasing, in conjunction with the overall performance and efficiency results presented, substantiates the intended meaning more clearly.

**<Comment #2-19>** *19. The training procedure depicted in figure 2 is in contrast with what has been mentioned as “End-to-End Training Framework”, Figure 2 is showing the backpropagation path back to only classifier module leaving out the MCMCE.*

**<Response #2-19>** We thank the reviewer for their observation regarding Figure 2 and its depiction of the training process in relation to our 'End-to-End Training Framework.' We believe there might have been a slight misunderstanding, possibly arising from focusing on only one of the two training paths illustrated in Figure 2.

Figure 2 is designed to compare two distinct training scenarios:

1. The Blue Path: This path illustrates the training process with our proposed MSMCE module. In this scenario, the input data first passes through the MSMCE module, and the output of MSMCE is then fed into the downstream classification model. Crucially, this blue flow does indeed extend back through the MSMCE module. This ensures that both the MSMCE module and the classifier are jointly optimized in an end-to-end manner.
2. The Red Path: This path illustrates the baseline training process without the MSMCE module, where raw (or conventionally preprocessed) data is directly fed into the classification model. In this scenario, naturally, the backpropagation path only updates the classifier module, as MSMCE is not part of this pipeline.

It appears the reviewer's comment might be referring to the red path, which accurately depicts a non-MSMCE baseline where backpropagation would indeed only affect the classifier. However, our claim of an 'End-to-End Training Framework' specifically pertains to the blue path, where MSMCE is an integral and learnable component of the overall model, and its parameters are updated via backpropagation from the final loss.

We hope this clarification, along with the existing descriptions in the manuscript, resolves the reviewer's concern.

**<Comment #2-20>** *20. “enabling an adaptive representation of the input.” the term “adaptive” has not been used correctly.*

**<Response #2-20>** Thank you for pointing out the potential misuse of the term 'adaptive' in this context. We agree that 'adaptive representation' might imply a dynamism or context-specific adjustment of the representation that is not the primary characteristic we intended to convey for the MSMCE module's output once trained.

Our intention was to highlight that the MSMCE module, through end-to-end training, learns a representation that is optimized for the specific task and dataset, rather than being a fixed, handcrafted representation. It automatically learns to represent the input in a way that is beneficial for the downstream classifier. To address this and use more precise terminology, we have revised the sentence in the **'Materials and Methods' section, at the end of the 'Training Process' subsection**. The revised sentence now reads: 'enabling a learned, data-driven representation of the input.'

We believe this phrasing more accurately describes the nature of the representation generated by our end-to-end trained MSMCE module.

**<Comment #2-21>** *21. 2.6 Data Processing Workflow: This section starts with a few paragraphs as its own introduction. These paragraphs need to be moved to the introduction or discussion depending on the context and in this section only the method should be discussed.*

**<Response #2-21>** Thank you for this feedback on the structure of the 'Data Processing Workflow' section. We agree that this section should focus solely on the methodology employed.

In the revised manuscript, we have streamlined the beginning of the 'Data Processing Workflow' section. We have removed the majority of the initial introductory and background paragraphs. Specifically, the discussion regarding the limitations of traditional feature engineering and the requirement for extensive prior knowledge has been relocated to the **'Discussion' section** where it provides better context. The 'Data Processing Workflow' section now directly describes the specific data processing steps applied in our study.

We believe these changes ensure that the section is now concise and strictly pertains to the methods used, as recommended.

**<Comment #2-22>** *22. The data processing section leaves most of the details out, referencing [2],[10]. details of the method should be outlined.*

**<Response #2-22>** We thank the reviewer for their feedback regarding the level of detail in our 'Data Processing Workflow' section. We apologize if our initial phrasing, such as 'we follow the approach described in [2],' was misconstrued as merely deferring to the cited literature without providing our specific implementation details. Our intention was always to describe the methods we employed, which are indeed based on the foundational work in references [2] and [10], and the text following these citations in our original manuscript was meant to outline these specifics.

To ensure greater clarity and directly address the reviewer's concern for a more explicit outline of the methods, we have **significantly revised and expanded the 'Data Processing Workflow' section** in our manuscript. This revision includes a more detailed, step-by-step description of how data from both LC-MS and SpiderMass datasets were processed, incorporating key parameters and methodologies inspired by or directly adopted from references [2] and [10]. **It is worth noting that after adjusting the citation order of the literature, the literature [2] becomes [9]**.

Specifically, the revised section now explicitly details:

1. For LC-MS Datasets (NSCLC, CRLM, RCC), aligning with Ref. [2], now [9]: We now clearly state the m/z binning width (0.1 Da), the specific 10-second RT window aggregation method (summing and averaging intensities per m/z bin within each window).
2. For SpiderMass Datasets (Canine Sarcoma), aligning with Ref. [10]: We detail the initial TIC filtering (threshold 1×10^4^), the 0.1 Da m/z binning (specifying the resulting feature count).

These elaborations, which can be found in the **'Data Processing Workflow' section of the revised manuscript**, provide a much more comprehensive account of our data processing pipeline. We believe this approach now sufficiently outlines the methods, enhances reproducibility, and resolves any previous ambiguity. We appreciate the opportunity to clarify our work.

**<Comment #2-23>** *23. Section 2.7- What is the strategy to divide data to training, validation, and test sets? Has k-fold strategy been used? How many times the model has been trained? What is the strategy to avoid overfitting? Are all the results related to a single (but same for all models) trial division? Or the random seed might be different for each tested model?*

**<Response #2-23>** Thank you for these important questions regarding our experimental setup and training strategy. We have revised Section 'Experimental Setup and Training Strategy' to provide a more comprehensive description.

1. Data Splitting Strategy: The overall dataset for each classification task was initially split into a training set (90%) and a fixed hold-out test set (10%). This initial split was performed using stratified sampling to maintain the original class proportions in both sets. The same random seed was used for this initial split across all experiments on a given dataset to ensure comparability. The 90% training set was then subjected to a k-fold cross-validation strategy for model training and validation.
2. K-Fold Cross-Validation: We employed a stratified k-fold cross-validation approach on the 90% training data. The number of folds was set to 6, and in each fold, the data was split into a training fold and a validation fold. The model was trained on the training fold and validated on the validation fold (primarily for early stopping and learning rate scheduling). To maintain comparison, the machine learning model was trained only on the training fold. The final reported performance metrics (Accuracy, Precision, Recall, F1-score) for each of the k models (one from each fold) were evaluated on the common, fixed 10% hold-out test set. The mean and standard deviation (95% bootstrap confidence intervals provided in supporting information S1) of these k test results are reported in our tables.
3. Number of Times Trained: For each specific model architecture (e.g., MSMCE -ResNet50) and dataset combination, the model was trained k times (once for each fold of the k-fold cross-validation).
4. Strategy to Avoid Overfitting
   a) Stratified sampling for all splits.
   b) Weight decay (L2 regularization) was applied in the Adam optimizer. An Early Stopping mechanism was employed based on validation loss to prevent excessive training.
   c) A Learning Rate Scheduler (ReduceLROnPlateau) was used to adjust the learning rate based on validation loss stagnation.
   d) Class weighting was applied to the CrossEntropyLoss function to handle potential class imbalances in the training folds.
5. Consistence of Trial Division and Random Seeds:
   a) All results for a given dataset are related to the single initial 90/10-train/test split, which was generated using a fixed random seed.
   b) The subsequent k-fold splits within the 90% training data were also controlled by the same random seed for StratifiedKFold, ensuring that for any given experiment configuration (dataset, model type), the exact same folds were used.

**<Comment #2-24>** *24. In the table 2,3 and 5 there are values for precision and F1 that are below 0.5. This does not make sense, because any model that has been trained on data should be better than a totally random model with 50% performance. Also, a value of exactly 0.5000 for recall seems not to be correct considering variability in the MS data.*

**<Response #2-24>** We thank the reviewer for their scrutiny of the performance metrics in Tables 2, 3, and 5. These specific low performance metrics for the original LSTM and Transformer models highlight a key motivation for our work: the significant challenge these architectures face when directly applied to very high-dimensional and sparse raw MS data without an effective feature representation or dimensionality reduction mechanism.

The observed performance, akin to random guessing (e.g., a recall of 0.5000 in a balanced binary task suggests the model might be consistently predicting one class or failing to discriminate effectively), indicates that these models, in their baseline configuration on the raw, high-dimensional input, likely failed to learn meaningful discriminative patterns from the data. The complexity and sparsity of the raw MS vectors, when fed directly into these sequence models without a tailored embedding like MSMCE, can lead to difficulties in optimization, convergence to poor local minima, or an inability to extract relevant features from the vast input space.

These results starkly contrast with the performance of the same LSTM and Transformer architectures when integrated with our MSMCE module, where all metrics significantly improve. This substantial improvement underscores the efficacy of MSMCE in transforming the raw MS data into a more learnable and informative representation for these models.

Therefore, these low baseline scores are presented not as an anomaly, but rather as an empirical demonstration of the difficulty certain advanced architectures have with raw, high-dimensional MS data, thereby emphasizing the significant contribution of our MSMCE module in making these architectures effective for such data.

**<Comment #2-25>** *25. Table 2 and 3 does not report number of trained models or any variability related to the performance. The absolute value shows enhancement when the MSMCE has been included. However, no significance statistical metric has been reported. The proper way would have been multiple trainings using multiple random divisions of trials and then reporting an average, std and a pvalue for each comparison.*

**<Response #2-25>** Thank you for your valuable feedback on the reporting of our experimental results in Tables 2 and 3. We have adopted your recommendations to provide a more robust statistical evaluation. In the revised manuscript, the performance metrics presented are now based on results from k-fold cross-validation (k=6), as detailed in **Section 'Experimental Setup and Training Strategy'**. This means each model architecture was effectively trained and evaluated multiple times. The mean performance values and their corresponding standard deviations are now directly included in the revised manuscript. Furthermore, to assess statistical significance, p-values from paired Wilcoxon signed-rank tests comparing models with and without MSMCE are referenced in the main text when discussing these comparisons. We believe these revisions fully address your concerns regarding the reporting of model repetitions, variability, and statistical significance.

**<Comment #2-26>** *26. “representing an increase of 1.608%”, this enhancement is not of value, unless repeated over several randomizations and then averaged.*

**<Response #2-26>** Thank you for this valuable feedback. We have taken the reviewer's suggestion into account to ensure the robustness of our reported performance enhancements. In the revised manuscript, all reported performance metrics, including the specific accuracy increase for ResNet-50 with MSMCE on the NSCLC dataset (Table 2), are now derived from experiments conducted using a k-fold stratified cross-validation, as detailed in the updated '**Experimental Setup and Training Strategy' section**. The presented accuracies are the mean accuracies obtained from these k folds, with each fold's model evaluated on the common hold-out test set. This approach directly addresses the need for results based on multiple evaluations.

**<Comment #2-27>** *27. Tables 2, 3,5 and 6 report performance for models LSTM and transformer, without providing details of architecture of each of these models. LSTM layers or transformer architecture could be used in a DL-based pipeline in many ways. Details need to be properly reported.*

**<Response #2-27>** Thank you for emphasizing the need for more specific architectural details for our LSTM and Transformer models. In the revised manuscript, we have concisely integrated the key architectures of both the baseline and MSMCE-enhanced versions of these models into the **'Training Process' subsection of the 'Materials and Methods' section**. This description includes how input spectra are processed into sequences. To maintain conciseness in the main text, we have not provided detailed model parameters therein but refer to our publicly available code for further implementation specifics. We believe this approach provides the necessary details while keeping the main text streamlined.

**<Comment #2-28>** *28. Table 6 (class12), precision and recall reported for multi-class arrangement. It is not clear if the final precisions the average of precisions for each class or a different strategy has been used.*

**<Response #2-28>** Thank you for the query regarding the calculation of precision and recall for multi-class results, specifically in Table 6. We have now added a clarifying statement in the **preliminary description of the 'Experiment and Results' section**, just before presenting the detailed tables. This statement clarifies that for all multi-class classification tasks reported, aggregate precision, recall, and F1-score metrics were computed using the **'macro' averaging strategy**. This means metrics were calculated for each class independently and then their unweighted mean was taken, ensuring each class contributes equally to the final average. We believe this placement and clarification effectively addresses how these metrics were derived for multi-class scenarios.

**<Comment #2-29>** *29. Figure 4, the reported accuracy drops at some epochs, which makes the reported accuracy metric to be questionless considering optimizers like Adam and loss functions like cross-entropy. How do the authors justify the drops?*

**<Response #2-29>** We thank the reviewer for carefully examining the training curves in Figure 4 and for raising this question about the observed drops in accuracy during some epochs.

The fluctuations, including temporary drops in validation accuracy observed in some of the ablation study curves in Figure 4 (particularly for models or configurations before the full MSMCE module is integrated, e.g., 'Baseline' or 'Encoder only'), are indeed an important observation. We attribute these drops primarily to the characteristics of the optimization process in the early stages of training, potentially influenced by the initial learning rate settings.

While the Adam optimizer and cross-entropy loss are generally robust, a relatively high initial learning rate, even with a decay scheduler, can sometimes cause the model to overshoot optima or explore regions of the loss landscape that temporarily decrease validation performance, especially on smaller or more complex datasets like the Canine Sarcoma (Classes=12) dataset used for this ablation study. As the learning rate is subsequently reduced by the 'ReduceLROnPlateau' scheduler (as described in our **'Experimental Setup and Training Strategy' section**), the optimization process tends to stabilize, leading to smoother convergence and more stable accuracy in later epochs. This is evidenced by the later parts of the curves in Figure 4, especially for the more complete model configurations (e.g., 'Channel Embedding' and 'Channel Concatenation' added), where accuracy generally becomes more stable and consistently high.

It is also worth noting that Figure 4 specifically depicts the ablation study where components are progressively added. The models with fewer components (e.g., the baseline ResNet-50 without any part of MSMCE, or with only the Encoder) might be more prone to such instabilities or suboptimal convergence paths compared to the full model integrating all parts of MSMCE, which consistently shows more stable and superior performance (as also seen in the final accuracy values in Table 7). The introduction of the Channel Embedding and Channel Concatenation components appears to contribute significantly to stabilizing the training and improving feature representation, leading to smoother accuracy curves, as also noted in the caption of Figure 4.

**<Comment #2-30>** *30. ResNet-50, DenseNet-121, and EfficientNet-B0 should be properly referenced.*

**<Response #2-30>** Thank you for this remainder. We have ensured that ResNet-50, DenseNet-121, and efficientNet-B0 are now properly referenced in the revised manuscript. The citations for these architectures have been added where these models are first mentioned in the **'Introduction' section and are included in the updated reference list**.

**<Comment #2-31>** *31. Figure 5, the lines should be transparent (alpha=0.8) for better visibility. The lines are blocking each other.*

**<Response #2-31>** Thank you for this helpful suggestion to improve the visibility of Figure 5. We have revised Figure 5 according to your recommendation, setting the transparency of the lines to reduce overlap and enhance clarity where multiple lines are present. The updated Figure 5 has been included in the revised manuscript.

**Reviewer #3**

**<Comment #3-1>** *1. Image Quality: The figures are currently of insufficient quality, making them unreadable. This significantly hinders the reader's ability to follow the results. I strongly recommend improving the resolution and clarity of all figures to ensure they are legible and informative.*

**<Response #3-1>** We apologize for the issues with the figure quality experienced by the reviewer. We believe this may be due to the compression of images when the journal generates the manuscript. We had initially submitted high-resolution figures. For this resubmission, we have re-checked and ensured that Figures 1 to 3 (and all other figures) are provided in high resolution, meeting PLOS ONE’s image guidelines, to ensure their legibility and clarity. We have also utilized the PACE tool as recommended to verify figure compliance.

**<Comment #3-2>** *2. Description of the Data: More detailed information about the dataset is necessary. Readers unfamiliar with this data may not understand its specific challenges, particularly in the context of classification tasks. Please elaborate on the nature of the dataset, potential difficulties, and how these may relate to the performance of the proposed representation technique?*

**<Response #3-2>** Thank you for this crucial feedback. We recognize the importance of providing a comprehensive understanding of the datasets, their inherent challenges, and how these factors relate to the performance and utility of our MSMCE module. We have substantially revised the manuscript to address this.

1. Enhanced Dataset Description: We have ensured that Table 1 now clearly presents core information for each dataset, including the specific mass spectrometry instrument used, the number of classes, the precise number of samples (after data processing) per class, and the m/z range covered.
2. Elaboration on Dataset Nature and Potential Difficulties: To visually illustrate the nature and potential difficulties of each dataset before applying MSMCE, we now discuss t-SNE visualizations of the processed input features. These plots (provided as S1 File in the Supporting Information) offer a qualitative insight into the initial class separability and complexity. For example, in the preliminary discussion of Experiment and Results section, we note that "Fig A in S1 File (Canine Sarcoma 12-class) illustrates significant overlap among several distinct sarcoma subtypes, underscoring the inherent challenges in achieving fine-grained classification... based solely on the original features." This helps readers understand the baseline difficulty.
3. Relating Dataset Challenges to MSMCE's Performance: We discuss how MSMCE’s encoder first performs a global embedding, effectively reducing dimensionality, and the subsequent channel embedding module creates a richer, multi-perspective representation. This is particularly relevant for all datasets, as their original spectrum dimension is very high. The improved performance with MSMCE suggests it makes this high-dimensional data more tractable for classifiers. When discussing datasets where t-SNE (S1 File) shows considerable initial class overlap, we now link the significantly improved classification accuracy achieved with MSMCE-enhanced models (Tables 5 & 6) to MSMCE's ability to learn more discriminative features that can better separate these intermingled classes. The multi-channel approach is posited to capture diverse underlying patterns crucial for this disentanglement.

We believe these comprehensive revisions to our dataset descriptions, the introduction of t-SNE visualizations to illustrate inherent challenges, and the explicit linking of these challenges to the demonstrated performance benefits of MSMCE, now provide a much clearer and more compelling narrative.

**<Comment #3-3>** *3. Error and Robustness Metrics: While the provided metrics illustrate the model’s performance, the lack of error analysis is a limitation. Including confidence intervals or other measures of uncertainty would provide a clearer picture of the method’s robustness. It would also facilitate a more rigorous comparison between methods.*

**<Response #3-3>** Thank you for highlighting the importance of error analysis and robustness metrics. In response to your feedback, we have incorporated these aspects into our revised manuscript. All performance metrics presented in our revised manuscript are now based on the outcomes of k-fold cross-validation (specifically, k=6), which provides multiple evaluations for each model on an independent hold-out test set. We now report the mean values for key performance metrics (Accuracy, Precision, Recall, and F1-score), and **Supplementary Information S1 details the 95% bootstrap confidence intervals for these mean metrics to quantify their uncertainty**. Furthermore, to facilitate more rigorous inter-method comparisons, when analyzing the performance improvements attributed to the MSMCE module in the manuscript, we reference **p-values from paired Wilcoxon signed-rank tests** to assess the statistical significance of the observed performance differences. We believe these additions to our statistical analysis more clearly demonstrate the robustness of our proposed method and provide robust support for rigorous comparisons between models, thereby fully addressing your concerns.

**<Comment #3-4>** *4. Comparison with Traditional Methods: The proposed technique is positioned as an improvement over traditional feature engineering methods. However, the manuscript does not clearly specify which traditional techniques were used to generate the “Original” baseline. Please provide more details about these baseline methods so readers can better assess the added value of MSMCE.*

**<Response #3-4>** We sincerely apologize for any previous miscommunication and lack of clarity in positioning our MSMCE module relative to traditional feature engineering methods. We thank the reviewer for pointing this out, as it has allowed us to significantly improve the framing of our work. We acknowledge that our manuscript may have inadvertently implied that MSMCE is a direct improvement or replacement for traditional, often biologically focused, feature engineering pipelines. This was an oversight on our part. Our data processing workflow and the MSMCE module itself operate under a different paradigm, one that aligns with and builds upon recent research (such as our cited works [2] and [10]) exploring the direct application of deep learning to raw or minimally processed mass spectrometry data for classification tasks.

To clarify:

1. **Different Aims and Outputs of Traditional Feature Engineering:** Traditional feature engineering in mass spectrometry analysis, particularly when aimed at biological interpretation or for use with classical machine learning, **typically involve processing a raw MS file into a structured quantitative/qualitative data table (often a .csv file)**. Such a data table is designed to reveal the types of compounds present in the original sample and their respective relative abundances or quantities. For instance, each row in the table might correspond to a compound or feature ion identified through specific rules (e.g., peak detection, deconvolution, database matching), while columns would contain its intensity, retention time, or other relevant attributes. This information is then used for subsequent biological insight generation or statistical analysis.
2. **Challenges in Direct Comparison due to Different Focus:** Our data processing approach, in contrast, focuses on an alternative pathway in MS data analysis: applying deep learning directly to raw or minimally processed (e.g., binned) spectra. For example, individual spectra from SpiderMass or RT-aggregated spectra from LC-MS (**where one original MS file can be processed into multiple MS instances**) are utilized. This differs significantly from traditional methods where one original file might primarily yield a single consolidated feature vector. Given these fundamental differences in processing paradigms and objectives, a direct, feature-by-feature comparison of MSMCE against a specific set of traditional feature engineering *techniques* (e.g., a pipeline of peak picking, alignment, and selection, ultimately generating one feature vector per original file) is challenging and not the core aim of our study.
3. Definition of 'Baseline' in Our Study: The 'Baseline' column in our tables refers to the application of models to MS data output by our data processing workflow (m/z binning, RT window aggregation, as described in the 'Data Processing Workflow' section).
4. Demonstrating MSMCE's Added Value: The added value of MSMCE is demonstrated by comparing the performance of the same deep learning classifier when it utilizes the representation generated by MSMCE versus when it processes the single-channel representation. The focus is on optimizing the input representation for these deep learning models.

The goal of MSMCE is to automatically learn a rich, multi-channel embedding to enhance the predictive performance of downstream deep learning models, rather than to produce features for direct biological interpretation of individual features in the traditional sense. Any previous statements in our manuscript highlighting the complexity of traditional feature engineering or the need for prior knowledge were intended to underscore the general challenges of MS data that motivate the exploration of representation learning, not to position MSMCE as a direct substitute for those specific traditional workflows.

We deeply regret any confusion caused by our earlier descriptions. We have thoroughly revised the manuscript, particularly in the **Introduction, the preliminary discussion of 'Experiment and Results', and the 'Discussion' section**. All statements that might have misleadingly positioned MSMCE as a direct improvement over traditional, biologically focused feature engineering have been rephrased. The revised text now consistently emphasizes that our work focuses on optimizing the feature representation of raw/minimally processed MS data specifically to improve the classification performance of deep learning models. Our 'Baseline' for deep learning models is clarified as these models directly processing the binned and normalized single-channel data, allowing for a clear assessment of MSMCE's additive value in enhancing this input representation.

We believe these comprehensive revisions now accurately reflect the scope and contribution of our research within the context of direct deep learning classification from MS data. Thank you again for guiding us to make these important clarifications.
