# Supplementary material for: MSMCE: A novel representation module for classification of raw mass spectrometry data
Source: PLoS One. 2025 Aug 6;20(8):e0321239. doi: 10.1371/journal.pone.0321239 (PMC12327681; doi:10.1371/journal.pone.0321239)
Supplement: Attachment — Submitted filename: Response_to_Reviewers_auresp_2.docx [file pone.0321239.s005.docx]

**Response letter for “MSMCE: A Novel Representation Module for Classification of Raw Mass Spectrometry Data”**

**(Original Paper Number: PONE-D-25-08088)**

Dear Editor and Reviewers,

Thank you for your letter dated June 19, 2025, and for the opportunity to revise our manuscript titled "MSMCE: A Novel Representation Module for Classification of Raw Mass Spectrometry Data" (PONE-D-25-08088). We also extend our sincere gratitude to the reviewers for their insightful comments and constructive suggestions, which have been invaluable in improving the quality and clarity of our paper.

We have carefully considered all the points raised by the Academic Editor and the reviewers. We believe that the revisions made have substantially strengthened the manuscript. We have addressed each comment in a point-by-point manner below, and for ease of review, we have highlighted the revisions in bold. The changes in the revised manuscript have been marked using the "Track Changes" feature in Microsoft Word, as requested. We have also uploaded a clean, unmarked version of the revised manuscript.

We hope that the revised manuscript is now suitable for publication in PLOS ONE.

Sincerely,

Fengyi Zhang, Boyong Gao, Yinchu Wang, Lin Guo, Wei Zhang, Xingchuang Xiong.

**CONTENTS**

[**Reviewer #2**	5](#_Toc201672601)

[**<Comment #2-1>**	5](#_Toc201672602)

[**<Comment #2-2>**	7](#_Toc201672603)

[**<Comment #2-3>**	10](#_Toc201672604)

[**<Comment #2-4>**	10](#_Toc201672605)

[**<Comment #2-5>**	11](#_Toc201672606)

[**<Comment #2-6>**	11](#_Toc201672607)

[**<Comment #2-7>**	12](#_Toc201672608)

[**<Comment #2-8>**	13](#_Toc201672609)

[**<Comment #2-9>**	13](#_Toc201672610)

[**<Comment #2-10>**	13](#_Toc201672611)

[**<Comment #2-11>**	14](#_Toc201672612)

[**<Comment #2-12>**	15](#_Toc201672613)

[**Reviewer #3**	16](#_Toc201672614)

[**<Comment #3-1>**	16](#_Toc201672615)

[**<Comment #3-2>**	16](#_Toc201672616)

**Reviewer #2**

**<Comment #2-1>** *1. The reported p-values appear inconsistent and potentially incorrect. All five instances report the exact same value (p = 0.03125), regardless of the magnitude of performance improvement. For example, in Table 5, the accuracy of the Transformer model on the Canine Sarcoma dataset increases markedly from 0.78 ± 0.00 to 0.99 ± 0.01 — a substantial improvement that would typically yield a much lower p-value (e.g., p < 0.01) if statistical significance were properly assessed. The uniformity of the reported p-values across different metrics and datasets raises concerns about the validity of the statistical testing procedure. Clarification is needed on how these p-values were computed and whether appropriate statistical tests were applied in each case.*

**<Response #2-1>** We sincerely thank the reviewer for their meticulous observation and for raising this important question regarding our p-value results. We completely understand why the recurrence of the exact same p-value (p = 0.03125) (**We rounded 0.03125 to 0.03 in the current manuscript**) across multiple instances would raise valid concerns about its validity. We would like to take this opportunity to clarify our statistical testing procedure. We confirm that all reported p-values were computed using the paired Wilcoxon signed-rank test on the performance scores from each of the 6 folds of our cross-validation (K=6). Therefore, the sample size for each test was N=6.

And most crucially, **the reason for this identical p-value appears across instances with different magnitudes of performance improvement is a direct consequence of the properties of the Wilcoxon signed-rank test itself**. As a non-parametric test, its calculation relies on the sign and ranks of the paired differences, not on the magnitude of these differences. In every instance where the p-value was reported as 0.03125, **it was the case that our proposed MSMCE-enhanced model outperformed its corresponding baseline model in all 6 folds of the cross-validation**. For a Wilcoxon signed-rank test with a sample size of N=6, the scenario where all 6 differences share the same sign (e.g., all positive) constitutes the most extreme and consistent positive result that the test can detect. In this "perfect sweep" situation, the test will always yield its minimum possible p-value, regardless of whether the performance improvement in each fold was marginal or substantial. For a two-sided test, this minimum p-value is precisely 0.03125. The detailed description is as follows:

1. **Test Setup and Hypotheses**

To clearly elaborate on our p-value calculation process, we detail here the specific steps of the **paired Wilcoxon signed-rank test** as applied in our case. As stated in the manuscript, we compare the paired performance scores (sample size N=6) of the "models integrated with MSMCE" against their "baseline" counterparts from the 6-fold cross-validation. The null hypothesis ($H_{0}$) for this test is that there is no difference in performance between the two models (i.e., the median of the performance differences is zero). Our two-sided test corresponds to the alternative hypothesis ($H_{a}$) that a difference in performance does exist (i.e., the median of the differences is not zero).

2. **Calculating the Test Statistic (**$\boldsymbol{W}$**)**

The calculation begins by taking the difference $d$ for each of the 6 pairs of performance scores. Subsequently, we rank the absolute values of these differences, $|d|$, from 1 to 6. The original signs (+ or -) are then reassigned to their corresponding ranks, and we separately calculate the sum of the positive ranks ($W^{+}$) and the sum of the negative ranks ($W^{-}$). Finally, the test statistic W is defined as the minimum of $W^{+}$ and $W^{-}$. The value of this $W$ statistic reflects the consistency of the difference between the two groups.

3. **Principle of the Two-Sided p-value Calculation**

The p-value represents the probability of observing a $W$ statistic as extreme as, or more extreme than (i.e., closer to 0), our calculated W, assuming the null hypothesis (no difference in model performance) is true. For a sample size of N=6, there are $2^{6}=64$ possible combinations of signs for the ranks. Because we are conducting a two-sided test, we consider extreme outcomes in both directions: a very small $W$ (indicating the MSMCE model is consistently better) and a very large $W$ (indicating the baseline model is consistently better). The p-value is the sum of the probabilities of these two extreme scenarios.

4. **Specific Calculation for p = 0.03125**

The recurring p-value of 0.03125 in our manuscript corresponds to the most extreme result possible for this test, where the test statistic W=0. This scenario occurs only when one condition is met: the model integrated with MSMCE outperforms the baseline model in every one of the 6 folds. In this case, all 6 performance differences are positive, making the sum of negative ranks (W−) equal to 0, the probability of its occurrence is $\left( 1/2 \right)^{6}=1/64$, and therefore the test statistic W is also 0m, the probability of its occurrence is $\left( 1/2 \right)^{6}=1/64$. For this "perfect sweep" situation, the two-sided p-value is calculated as: (Probability of all differences being positive) + (Probability of all differences being negative) = (1/64) + (1/64) = 2/64 = 0.03125. This explains why this fixed, minimum p-value is obtained whenever a consistent improvement is observed, regardless of the magnitude of that improvement.

Therefore, the uniformity of the p-value is not an artifact of an incorrect procedure. On the contrary, it serves as strong evidence that the performance enhancement from our proposed MSMCE module is exceptionally robust and consistent across all data splits in those specific comparisons.

**<Comment #2-2>** *2. As noted in the previous round, the performance values reported in Tables 2, 3, and 4 — such as 0.25, 0.33, and 0.4 — are notably lower than what would be expected from a random classifier in a binary classification task. This suggests that the number of model training iterations may have been insufficient to yield a reliable evaluation. The authors report using 6-fold cross-validation (k=6); however, this was applied to a single 90/10 train-test split, meaning the same data partitioning was used throughout the evaluation (i.e., n=1). Given the class imbalance evident in the t-SNE plots provided in the Supplementary Materials, repeating the 90/10 split multiple times is essential to ensure a fair comparison between the baseline and enhanced models. Each repetition can be followed by k-fold cross-validation (potentially with a lower k if computational feasibility is a concern). This approach helps mitigate the impact of any train-test split, smoothing out performance fluctuations caused by randomness in the data. For instance, a low score such as 0.25 from one split may be balanced by higher scores in others, leading to a more representative average.*

**<Response #2-2>** We sincerely thank the reviewer for this insightful and important comment. The concern regarding whether the low performance values (e.g., 0.25, 0.33, 0.4) were artifacts of a specific data split is entirely valid. A more robust validation protocol involving multiple independent splits is crucial for ensuring the reliability of our conclusions.

To that end, we have diligently followed your valuable recommendation and conducted a new, more comprehensive set of validation experiments. Specifically:

1. **Multiple Independent Splits**: We focused on the baseline Transformer model's performance on the NSCLC, CRLM, and RCC datasets. We repeated the 90/10 train-test split using three different random seeds (3407, 42, and 1234).
2. **Nested Cross-Validation**: For each of these independent splits, we subsequently performed a full 6-fold cross-validation on the corresponding training set.

NSCLC

| Seed=3407 | Fold | Accuracy | Precision | Recall | F1 Score |
| --- | --- | --- | --- | --- | --- |
|  | 1 | 0.517062 | 0.258531 | 0.5 | 0.340831 |
|  | 2 | 0.482938 | 0.241469 | 0.5 | 0.325663 |
|  | 3 | 0.517062 | 0.258531 | 0.5 | 0.340831 |
|  | 4 | 0.482938 | 0.241469 | 0.5 | 0.325663 |
|  | 5 | 0.482938 | 0.241469 | 0.5 | 0.325663 |

| Seed=42 | Fold | Accuracy | Precision | Recall | F1 Score |
| --- | --- | --- | --- | --- | --- |
|  | 1 | 0.490457 | 0.245228 | 0.5 | 0.329065 |
|  | 2 | 0.490457 | 0.245228 | 0.5 | 0.329065 |
|  | 3 | 0.509543 | 0.254772 | 0.5 | 0.337548 |
|  | 4 | 0.509543 | 0.254772 | 0.5 | 0.337548 |
|  | 5 | 0.490457 | 0.245228 | 0.5 | 0.329065 |

| Seed=1234 | Fold | Accuracy | Precision | Recall | F1 Score |
| --- | --- | --- | --- | --- | --- |
|  | 1 | 0.484095 | 0.242047 | 0.5 | 0.326189 |
|  | 2 | 0.515905 | 0.257953 | 0.5 | 0.340328 |
|  | 3 | 0.484095 | 0.242047 | 0.5 | 0.326189 |
|  | 4 | 0.484095 | 0.242047 | 0.5 | 0.326189 |
|  | 5 | 0.484095 | 0.242047 | 0.5 | 0.326189 |

CRLM

| Seed=3407 | Fold | Accuracy | Precision | Recall | F1 Score |
| --- | --- | --- | --- | --- | --- |
|  | 1 | 0.49542 | 0.24771 | 0.5 | 0.331292 |
|  | 2 | 0.49542 | 0.24771 | 0.5 | 0.331292 |
|  | 3 | 0.50458 | 0.25229 | 0.5 | 0.335362 |
|  | 4 | 0.50458 | 0.25229 | 0.5 | 0.335362 |
|  | 5 | 0.49542 | 0.24771 | 0.5 | 0.331292 |

| Seed=42 | Fold | Accuracy | Precision | Recall | F1 Score |
| --- | --- | --- | --- | --- | --- |
|  | 1 | 0.500694 | 0.250347 | 0.5 | 0.333642 |
|  | 2 | 0.499306 | 0.249653 | 0.5 | 0.333025 |
|  | 3 | 0.500694 | 0.250347 | 0.5 | 0.333642 |
|  | 4 | 0.499306 | 0.249653 | 0.5 | 0.333025 |
|  | 5 | 0.500694 | 0.250347 | 0.5 | 0.333642 |

| Seed=1234 | Fold | Accuracy | Precision | Recall | F1 Score |
| --- | --- | --- | --- | --- | --- |
|  | 1 | 0.494588 | 0.247294 | 0.5 | 0.330919 |
|  | 2 | 0.505412 | 0.252706 | 0.5 | 0.33573 |
|  | 3 | 0.505412 | 0.252706 | 0.5 | 0.33573 |
|  | 4 | 0.494588 | 0.247294 | 0.5 | 0.330919 |
|  | 5 | 0.505412 | 0.252706 | 0.5 | 0.33573 |

RCC

| Seed=3407 | Fold | Accuracy | Precision | Recall | F1 Score |
| --- | --- | --- | --- | --- | --- |
|  | 1 | 0.314341 | 0.15717 | 0.5 | 0.239162 |
|  | 2 | 0.685659 | 0.34283 | 0.5 | 0.40676 |
|  | 3 | 0.685659 | 0.34283 | 0.5 | 0.40676 |
|  | 4 | 0.685659 | 0.34283 | 0.5 | 0.40676 |
|  | 5 | 0.685659 | 0.34283 | 0.5 | 0.40676 |

| Seed=42 | Fold | Accuracy | Precision | Recall | F1 Score |
| --- | --- | --- | --- | --- | --- |
|  | 1 | 0.321383 | 0.160691 | 0.5 | 0.243217 |
|  | 2 | 0.678617 | 0.339309 | 0.5 | 0.404272 |
|  | 3 | 0.678617 | 0.339309 | 0.5 | 0.404272 |
|  | 4 | 0.678617 | 0.339309 | 0.5 | 0.404272 |
|  | 5 | 0.678617 | 0.339309 | 0.5 | 0.404272 |

| Seed=1234 | Fold | Accuracy | Precision | Recall | F1 Score |
| --- | --- | --- | --- | --- | --- |
|  | 1 | 0.68694 | 0.34347 | 0.5 | 0.407211 |
|  | 2 | 0.68694 | 0.34347 | 0.5 | 0.407211 |
|  | 3 | 0.68694 | 0.34347 | 0.5 | 0.407211 |
|  | 4 | 0.68694 | 0.34347 | 0.5 | 0.407211 |
|  | 5 | 0.68694 | 0.34347 | 0.5 | 0.407211 |

The results from these new experiments (3 independent splits × 6 folds = 18 runs), are highly consistent with our original findings. This strongly indicates that the observed low scores are not an artifact of a specific data partition. **Rather, they genuinely reflect the inherent challenge and insufficient representational power of the standard Transformer model when tasked with directly processing such high-dimensional and complex raw mass spectrometry data.**

To further elucidate and validate this conclusion, we investigated the underlying reason for this poor performance. We hypothesized that these extremely low scores are due to the model entering a state of "Mode Collapse"—where it ceases to learn and instead predicts a single class for all samples. To provide direct evidence for this, **we have now added confusion matrices for these low-performing baseline models to the Manuscript**. These confusion matrices clearly visualize the model's extremely skewed predictions, offering intuitive proof that mode collapse has occurred.

In summary, we are very grateful for your suggestion. It has not only pushed us to strengthen the reliability of our study with more rigorous validation, but has also led us to provide a deeper and clearer explanation for the baseline models' performance bottleneck through the inclusion of confusion matrices.

**<Comment #2-3>** *3. The p-values in the tables appear to be reported selectively and not consistently across all model comparisons. Specifically, only three p-values are provided: the accuracy of ResNet-50 on the NSCLC dataset, the accuracy of DenseNet-121 on the CRLM dataset, and the F1-score of DenseNet-121 on the RCC dataset. Reporting statistical significance solely for accuracy — without corresponding significance measures for other relevant metrics such as precision or recall — provides an incomplete assessment of model performance. This is particularly concerning in imbalanced classification tasks, where improvements in accuracy may mask critical deficiencies, such as an increase in false positives or false negatives. To ensure scientific rigor and transparency, all key performance metrics reported in the tables — especially those being used to support claims of improvement — should be accompanied by appropriate statistical tests and corresponding p-values. Furthermore, if a reported result is not statistically significant, it should be clearly labeled as such or omitted to avoid misinterpretation.*

**<Response #2-3>** We sincerely thank the reviewer for raising this extremely important point. We completely agree that comprehensive and consistent reporting of statistical test results is crucial for ensuring the rigor and transparency of our research, especially when dealing with imbalanced datasets. To systematically address the issues you have raised and to improve the clarity of our manuscript, we have made the following revisions:

1. **Centralized Description of p-value Calculation:** We have added a detailed paragraph describing our statistical analysis methodology in the **introductory section of the "Results" section**. This description clarifies that all p-values were uniformly calculated using a two-sided, paired Wilcoxon signed-rank test (N=6) on the results from our 6-fold cross-validation. This ensures the uniformity and transparency of our statistical methods.
2. **Streamlined and Unified Table Content:** To improve the readability and aesthetic presentation of the tables, while focusing on the most representative and comprehensive evaluation metrics, we have streamlined all performance comparison tables (Tables 2-6) in the manuscript. We have **removed the display of Precision and Recall, retaining only Accuracy and F1-Score, which we believe are sufficient to reflect the overall performance of the models**.
3. **Comprehensive Inclusion of Key Metric p-values:** In the streamlined tables, for every Accuracy and F1-Score corresponding to the MSMCE-integrated models, we have now included the **p-value from the comparison against the respective baseline model**. This ensures that all key performance metrics used to support our claims of improvement are accompanied by their corresponding statistical evidence.
4. **Explanation for Non-significant Results:** For any results where the performance improvement was found to be not statistically significant (p ≥ 0.05), we have now included explicit discussions and explanations in the main text of the manuscript. We discuss the potential reasons for this, **such as the baseline model already reaching a performance ceiling, thereby avoiding any potential misinterpretation**.

We believe that with these revisions—namely, a centralized methodological description, comprehensive p-value reporting for key metrics in tables, and discussion of non-significant results in the text—we have fully and rigorously addressed your concerns. Thank you again for your valuable feedback, which has greatly improved the quality of our manuscript.

**<Comment #2-4>** *4. Line 646: “FLOPs of all these models significantly decrease”, the p-value of the significance, the test name, and the sample size for the test are missing for this claim.*

**<Response #2-4>** We thank the reviewer for this sharp observation. We would like to clarify that Floating Point Operations (FLOPs) is a deterministic metric, calculated from a fixed model architecture and input size. For any given model, this calculation yields a single, constant value, not a distribution of results from multiple runs. As there is no statistical variance associated with this metric, performing a significance test (such as a p-value test) is not applicable.

To correct our improper phrasing and present the findings more accurately, we have made the following revisions:

1. **We have removed the subjective term "significantly" from the sentence.**
2. **We have revised the statement to be an objective, quantitative description of the reduction, guiding the reader to the figure for specific values.**

The revised sentence are as follows: " However, after being integrated with the MSMCE module, the FLOPs of all these models are substantially reduced (as detailed in **S2**), and the model sizes also decrease, while classification accuracy improves considerably."

**<Comment #2-5>** *5. In the supporting material, the dots on the figures need to be transparent and with a narrow margin for each marker. The points are obstructing each other.*

**<Response #2-5>** We sincerely thank the reviewer for their valuable suggestions on improving the t-SNE visualizations in the supplementary materials. Following your recommendations, we have redrawn all the t-SNE figures, setting the data points to be semi-transparent and giving them narrower margins by reducing the marker edge width. This ensures that the individual points are now much clearer.

Furthermore, we have **moved the figure from the supplementary materials into the main manuscript, placing it within the "Data Processing Workflow" section**. We believe this change will provide readers with an earlier and more direct visual understanding of each dataset's characteristics and the challenges we address.

Thank you again for your constructive feedback, which has helped us improve the quality and clarity of our manuscript's figures.

**<Comment #2-6>** *6. The test for 1 p-value has been reported as signrank test but others are missing the statistical test name. Adding a sentence to clarify that the same test has been used to calculate all significance levels could help clarify. Please also include number of samples used to calculate p value (probably k=6).*

**<Response #2-6>** We sincerely thank the reviewer for pointing out the inconsistency in our reporting of statistical tests. We completely agree that providing clear and consistent information about the test method and sample size is crucial for the clarity and reproducibility of our research. To address this comprehensively, we have implemented a two-part revision to ensure that our statistical methodology is both rigorously defined and consistently reported throughout the manuscript:

1. **Centralized Definition in the "Experiment and Results" Section:** Following your suggestion, we have added a paragraph to the introductory part of our "Experiment and Results" section. This paragraph now serves as a central definition, clarifying that all statistical comparisons were performed using the paired Wilcoxon signed-rank test with samples derived from our 6-fold cross-validation (N=6). This ensures that readers understand the uniform standard for our statistical validation before encountering any specific results.
2. **Standardized Reporting with Sample Size in Results:** Based on this central definition, we have standardized the reporting format for p-values within the results. As you recommended, we now consistently include the sample size (N=6) with each reported p-value. For example, the reporting of a key result now reads: "As shown in Table 2, on the NSCLC dataset, MSMCE-ResNet50 achieved an accuracy of 0.9785, a statistically significant increase of 1.28% (p=0.03, N=6) compared to the original model accuracy of 0.9661."

We believe this approach fully addresses all points of the reviewer's concern and significantly improves the scientific rigor and clarity of our manuscript. Thank you again for your constructive guidance.

**<Comment #2-7>** *7. Line 542-546, Lines 542–546 — The term “training crash” is not clearly defined or quantified, making it difficult to interpret its impact or relevance. I recommend moving lines 542–554 to the Discussion section, as this content is largely descriptive and does not report any measurable or validated performance metrics. Relocating this section would improve the logical structure of the Results section by keeping it focused on objective findings.*

**<Response #2-7>** We sincerely thank the reviewer for pointing out our inappropriate choice of words. Our use of the term “training crash” was imprecise and ill-defined, failing to accurately describe the experimental phenomenon we observed. We appreciate your guidance and have followed your recommendation to both clarify this phenomenon with a more precise description and relocate the paragraph to the more appropriate Discussion section.

Our specific revisions are as follows:

1. **Relocated the Paragraph:** We have moved the paragraph from the “Results” section to the “Discussion” section to improve the paper's logical structure.
2. **Corrected Improper Wording with Technical Terms:** We have clarified that our improper term “training crash” actually refers to the model failing to learn meaningful features and instead predicting a single class for all samples—a phenomenon commonly known in machine learning as **"Mode Collapse" or "Converging to a trivial solution."** This mode of training failure is quantitatively reflected in the poor performance metrics of the baseline Transformer model in the binary classification tasks (e.g., an F1-score of approximately 0.33 in Table 2-4).

**<Comment #2-8>** *8. Line 547- Line 547 — The use of the term “excellent” to describe the model’s performance is not appropriate in the context of a scientific report. Descriptive terms such as this are subjective and should be replaced with objective, metric-based statements that accurately reflect the quantitative results.*

**<Response #2-8>** Thank you for this valuable feedback. We completely agree that subjective descriptors such as "excellent" are inappropriate in a scientific report and that performance should be described using objective, metric-based statements. To make our statement more rigorous and quantitative, we have revised the original sentence to replace the subjective evaluation with a specific, comparative description of the model's performance.

The revised sentence is as follows:

**"It is noteworthy that on the NSCLC dataset, the RF model achieved the highest F1-score of 0.9935 among all tested models."**

**<Comment #2-9>** *9. Line 580, Thie seems to be a typo.*

**<Response #2-9>** Thank you for your keen observation and careful correction. "Thie" was indeed a typo and should be "The". We have corrected this error in the revised manuscript.

**<Comment #2-10>** *10. Line 433 Using the term Experimental is not appropriate for this section. Similarly line 489. The model fitting and testing has been performed in silico and no experiment was performed in a lab.*

**<Response #2-10>** We sincerely thank the reviewer for this precise point on terminology. To accurately reflect the computational nature of our work, we have revised the section headings as per your suggestion to improve the manuscript's rigor and clarity. The specific changes are as follows:

1. **The "Experimental Setup and Training Strategy" has been changed to " Training Strategy".**
2. **The "Experiment and Results" has been changed to "Results".**

We believe these new headings more accurately describe our research. Thank you again for your valuable guidance.

**<Comment #2-11>** *11. In figure 4, the dashed lines are reporting accuracy over “val”. However, the term “val” needs to be replaced with “test”, as no k-fold validation has been used for the ablation study, and the performance is measured over test set. Similarly, the term “validation performance” needs to be corrected as “performance over the test set”.*

**<Response #2-11>** We would like to thank the reviewer for their meticulous review of our figures and for raising this important point about terminology. We fully understand the importance of making a clear distinction between the "validation set" and the "test set", and we would like to take this opportunity to clarify our experimental methodology and explain why the use of "validation" in the figure is appropriate.

The test set should only be used for the final performance evaluation after the model has completed all training epochs. We have strictly adhered to this principle in our experiments. In our study, the dataset was divided into three distinct sets: a training set, a validation set, and a test set.

1. **Training Set:** This set is used to compute the gradients and update the model's weights during each epoch.
2. **Validation Set:** This set is held out from the training process. Its purpose is to evaluate the model's performance at the end of each epoch. This allows us to monitor the training process, diagnose potential overfitting, and ultimately select the "best" model based on its performance (e.g., the lowest validation loss or highest validation accuracy).
3. **Test Set:** This set is completely isolated from the training and validation processes. It is used only once, after the entire training procedure is finished, to assess the final generalization ability of the selected "best" model. Consequently, we only obtain a single, final performance score for the test set, not a curve that varies with epochs.

As the reviewer observed, the x-axis of Figure 4 represents the training epoch. The figure is intended to show the performance dynamics of different model variants during the ablation study's training process. Therefore, the dashed line in the plot correctly reports the accuracy fluctuation calculated on the validation set on an epoch-by-epoch basis. This is a standard practice in the deep learning field, aiming to visually compare the convergence speed and stability of models under different settings.

We assure the reviewer that the test set was only used to report the final results in our tables and was evaluated only once after all epochs were completed. The curves in Figure 4 reflect the performance on the validation set, which is crucial for model selection.

Thank you again for your valuable feedback.

**<Comment #2-12>** *12. Line 644, abbreviation of term FLOP has been repeated twice.*

**<Response #2-12>** Thank you for your careful review. We acknowledge that the abbreviation for "FLOPs" was redundantly defined. We have removed the second instance in the revised manuscript to ensure it is defined only once.

**Reviewer #3**

**<Comment #3-1>** *1. Inclusion of Supplementary Figures (A to C): The t-SNE visualizations currently provided in the Supplementary Information (Figures A to C) offer valuable insights into the intrinsic structure and difficulty of the classification tasks associated with each dataset. If feasible, I recommend incorporating these figures into the main manuscript to strengthen the presentation of the dataset characteristics and facilitate the reader’s understanding of the challenges involved.*

**<Response #3-1>** We sincerely thank the reviewer for this excellent suggestion. As per your recommendation, we have moved the t-SNE figures (formerly Supplementary Figures A to C) from the Supplementary Information into the main body of the manuscript. They are now **located within the "Data Processing Workflow" section**. We believe this change allows readers to gain an immediate visual context of the data structure, which facilitates a better understanding of our work's background and significance.

Thank you again for this constructive feedback, which has significantly improved the clarity and presentation of our paper.

**<Comment #3-2>** *2. Presentation of Bootstrap Confidence Intervals: Supplementary Table S3, which reports 95% bootstrap confidence intervals for the mean performance metrics, provides important information on the robustness of the results. I encourage the authors to consider including these intervals directly in the main performance tables of the manuscript (e.g., Tables 2 to 6), so that the uncertainty associated with each metric is readily visible to the reader.*

**<Response #3-2>** We sincerely thank you for the valuable suggestion to incorporate the 95% bootstrap confidence intervals directly into the main performance tables.

In this revision, we received feedback from other reviewer emphasizing the need for enhanced statistical rigor, particularly by providing significance testing for the performance improvements. To directly address this primary concern, we have chosen to add the corresponding p-values for our key metrics (Accuracy and F1-Score) in the main performance tables (Tables 2-6). We believe the p-value most directly answers the critical question of whether the observed improvements are statistically significant.

We considered that including both p-values and confidence intervals after each metric would make the tables quite crowded, potentially compromising their readability and aesthetic presentation. Therefore, to strike a balance between providing comprehensive information and maintaining a concise format, we have decided on the following approach:

1. We will prioritize the display of p-values in the main tables to highlight statistical significance.
2. We will continue to present the complete 95% bootstrap confidence intervals in Supporting Information S2 for readers who wish to delve deeper into the robustness of the results.

We believe this arrangement fulfills the core requirement for statistical significance while still making all information regarding the robustness of our findings fully available via the supplementary materials. Thank you very much for your suggestion, which has encouraged us to think more deeply about how to best present our data with clarity.
